# Efficacy of psychotropic medications on suicide and self-injury: a meta-analysis of randomized controlled trials

**DOI:** 10.1038/s41398-022-02173-9

**Published:** 2022-09-21

**Authors:** Xieyining Huang, Lauren M. Harris, Kensie M. Funsch, Kathryn R. Fox, Jessica D. Ribeiro

**Affiliations:** 1 Department of Psychology, Florida State University, Tallahassee, FL USA; 2 School of Behavioral and Brain Sciences, University of Texas at Dallas, Richardson, TX USA; 3 Department of Psychology, University of Denver, Denver, CO USA

**Keywords:** Psychology, Pharmacology

## Abstract

Using psychotropic medications to treat and prevent self-injurious thoughts and behaviors (SITBs) has become increasingly popular, but conclusive evidence supporting the efficacy this approach remains elusive. To empirically examine whether psychotropic medications are efficacious treatments for SITBs, the present meta-analysis comprehensively summarizes all published randomized controlled trials (RCTs) that have reported the causal effects of psychotropic medications on suicide and self-injury. A total of 251 papers from 718 unique RCTs were included. A frequentist pairwise approach was adopted for meta-analyses. Potential effect modifiers were examined via met regressions and potential biases were evaluated through sensitivity analyses. On average, medications yielded an 8% reduction in SITB frequency and a reduction of 0.2 standard deviations in symptoms and severity. Findings were largely consistent across potential effect modifiers, and significant evidence of publication bias was not detected. Only one medication class (i.e., antipsychotics) and two specific medications (i.e., citalopram, ketamine) produced larger-than-average treatment effects. Psychostimulants and typical antipsychotics may produce iatrogenic effects. Less than 4% of included studies required individuals to exhibit SITBs, and nearly half of analyzed effects were drawn from studies that excluded individuals on the basis of SITB risk. Taken together, findings suggest that psychotropic medications produce small treatment effects on SITBs; however, these findings should be considered in light of the methodological constraints of the existing literature, including the lack of studies intentionally including individuals with SITBs. It is critical for future RCTs to prioritize including individuals with existing SITBs to further clarify treatment effects in self-injurious and suicidal populations. Additional research is needed to better understand the treatment mechanisms of psychotropic medications and identify the causal processes underlying SITBs.

Using psychotropic medications to treat and prevent self-injurious thoughts and behaviors (SITBs) has become increasingly popular, but conclusive evidence supporting the efficacy this approach remains elusive. As SITB rates rise [1, 2], it is critical to establish whether psychotropic medications are efficacious treatments for SITBs.

Multiple reviews and meta-analyses [3–5] have endeavored to address this important knowledge gap; however, prior research in this domain has typically focused on one or a few medications, examined their efficacy within the context of specific disorders or populations, or examined one or a few SITB outcomes. These narrower summaries are valuable for specialized questions such as, *Is Lithium more efficacious than placebo in preventing suicide in bipolar disorder?* However, they are ill-suited to address broad questions such as, *Which psychotropic medication is the most efficacious for preventing suicide?* To establish whether psychotropic medications are efficacious for treating and preventing STIBs, a comprehensive meta-analysis is needed.

The present meta-analysis empirically addresses this knowledge gap by providing a quantitative synthesis of all published randomized controlled trials (RCTs) reporting the causal effects of psychotropic medications on SITBs. Our specific questions of interest are: 1) What is the overall efficacy of psychotropic medications on SITBs, and does efficacy depend on outcome type (i.e., type of SITB)? 2) Are some medications better than others at treating and preventing SITBs? and 3) What moderates the efficacy of psychotropic medications?

The present study represents a planned extension of a meta-analytic effort aimed at understanding the broad trends of SITB interventions [6]. In this effort, we provide a deep investigation of the state of the science regarding the efficacy of psychotropic medications for treating and preventing SITBs. This specific focus allows us to conduct detailed analyses to ascertain what type of medications may be the most beneficial for suicide and self-injury, and to explore how different types of psychotropic medications may act differently for different populations. Such information is critical for clinicians treating individuals with SITBs.

Several patterns of findings are possible. Perhaps no psychotropic medication is particularly efficacious for addressing SITBs. If so, patients may benefit from receiving other interventions while new medications are developed. Or, perhaps only certain medications are efficacious for SITBs. If so, prioritizing dissemination of the few efficacious medications may be beneficial. Perhaps most medications produce small effects on SITBs, indicating that interventions which are low-cost, easier to administer, and with fewer side effects should be prioritized. Or perhaps many medications are highly efficacious, but other constraints (e.g., adherence) may have prevented their effects from being reflected in SITB rates. If so, research should examine how to improve treatment access and compliance.

We expected moderate overall treatment effects, with stronger effects for medications designed to treat more severe symptoms (e.g., psychosis). If some medications outperform others, this would provide clear direction for future research, dissemination, and implementation efforts. If all medications produce similar effects, however, this could signal the need for fundamental changes in the way that we understand and develop medications for SITBs. Because there are many factors that might potentially influence the efficacy of psychotropic medications, moderator analyses may help determine the conditions under which medications are most efficacious.

Findings from the present study may enhance our ability to identify which medications are most efficacious for whom and for what SITB outcomes. They may also shed light on areas that require further research efforts to develop better interventions. Ultimately, we hope that our findings will facilitate reductions in SITB rates as research efforts are concentrated on areas with the most urgent needs.

## Methods

The present study extends a prior meta-analysis which evaluated the efficacy of a variety of SITB interventions [6]. Analyses were conducted using the dataset published by Fox et al. [6], which was updated to include newly published articles. Many critical questions pertaining to psychotropic medication efficacy were outside the scope of the original Fox et al. (2020) effort, and consequently remain unaddressed. The present effort takes steps toward addressing this gap, evaluating which medications are most beneficial for SITBs and how different medications may uniquely affect different populations.

### Search process

We updated a search [6] identifying RCTs published in English in print or online before January 1, 2018 to include papers published before January 1, 2021. Databases included PubMed, PsycINFO, Google Scholar, and ClinicalTrials.gov. Search terms included “treatment”, “intervention”, “therapy”, “suicide”, “self-injury”, “self-directed violence”, “self-harm”, “self-mutilation”, “self-cutting”, “self-burning”, and “self-poisoning”. Reference sections of reviews and meta-analyses emerging from this search were reviewed.

### Inclusion and exclusion criteria

An RCT design (i.e., random assignment to a treatment or control condition) was required. Interventions were not required to target SITBs, but an assessment of the occurrence or severity of SITBs *post* treatment was required. Interventions (i.e., treatment conditions) of interest were required to involve psychotropic medications. Studies that adopted an RCT design but did not assess the effects of interventions involving psychotropic medications were excluded. Studies that only included one arm with psychotropic medications (e.g., SSRIs compared with psychotherapy) were excluded as these studies do not facilitate comparisons among medications.

Papers that only reported outcomes relevant to SITBs (e.g., willingness to help peers experiencing SITBs) but did not examine the occurrence, frequency, or severity of SITBs were excluded. In a similar vein, papers that solely reported merged outcomes combining SITBs with other outcomes (e.g., gambling, violence towards others) were excluded. Studies lacking necessary information were excluded if contacted authors could not provide data.

Because we were interested in evaluating the overall efficacy of psychotropic medications on SITBs, we did not specify inclusion or exclusion criteria based on sample characteristics such as demographics (e.g., age) or clinical severity (e.g., prior history of suicidal behaviors, comorbid diagnoses). Instead, various study and sample characteristics were evaluated as potential effect modifiers (see *Data Analysis* for additional details).

### Data extraction

Data extraction and coding procedures have been described in detail previously [6]. Briefly, all treatment effects were coded by coauthors and trained research assistants on the dimensions delineated below, and all discrepancies were discussed and resolved by consensus.

When a paper reported multiple RCTs, statistics were extracted from each RCT whenever possible; otherwise, aggregated statistics were extracted. Most studies reported treatment effects on multiple SITB outcomes, which may result in dependence in included effect sizes and could lead to slight underestimation of effect sizes [7]. To reduce the likelihood of this possibility, and to avoid redundancy across articles published from the same trials, only unique effect sizes from each article were extracted.

#### Author, year, and era

Authors and publication year were extracted. The publication era was categorized via 10-year intervals.

#### SITB outcomes

Outcomes included: (1) suicide ideation or plan; (2) suicide attempt (i.e., intentional self-injury with nonzero intent to die); (3) suicide death (i.e., suicide attempt resulting in death); (4) NSSI (i.e., intentional self-harm without intent to die); (5) self-harm (i.e., intentional self-harm regardless of suicidal intent); (6) other/combined SITBs (e.g., suicidal gestures, outcomes combining suicide ideation and attempt). As stated above, outcomes that lumped a SITB outcome with a non-SITB related outcome (e.g., self-harm and risky sexual behavior) were excluded.

Of note, some outcomes were reported in binary form (e.g., presence or absence of suicidal ideation) whereas others were reported as continuous outcomes (e.g., scale score indicating severity of suicidal ideation). These outcomes are considered separately in analyses; odds ratios are the primary effect size for binary outcomes, and standardized mean differences are the primary effect size for continuous outcomes. Given that fewer than 5% of the pairwise comparisons reported results using continuous measures, analyses of continuous outcomes were limited to examining the effects of each psychotropic medication on aggregated continuous SITB outcomes, rather than each continuous SITB outcome separately.

#### Sample severity

Samples recruited based on SITB history were labeled “self-injurious”. Samples required to exhibit psychopathology but not to have a SITB history were considered “clinical”. Clinical samples were further coded as either “SITB-excluded” (i.e., participants were excluded if they were deemed to be at risk of SITBs) or “not SITB-excluded” (i.e., participants were not excluded based on putative SITB risk). Samples not required to exhibit SITBs or psychopathology (e.g., samples receiving interventions prophylactically) were considered “general”.

#### Sample age

We extracted mean sample age and age group at baseline: (1) child/adolescent (i.e., all participants under 18); (2) mixed adolescent and adult (i.e., participants below and above 18); (3) adult (i.e., all participants at least 18 but below 65); (4) older adult (i.e., all participants at least 65); (5) mixed adult and older adult (i.e., all participants above 18 but only some over 65); and (6) all age groups.

#### Sample sex

We extracted the percentage of female participants.

#### Sample size

Sample size at the time of randomization was extracted.

#### Sample race

When available, we extracted information regarding the race of individuals included in each sample.

#### Intervention target

Based on the primary treatment aim reported in the included studies, we coded treatments as targeting either psychopathology broadly defined or SITBs specifically.

#### Intervention components

Interventions which solely involved psychotropic medication were coded as “medication only”. If interventions included additional components (e.g., psychotherapy), these components were specified (e.g., “medication + psychotherapy”).

#### Blind status

Trials were coded as single blind, double blind, or not blind. Not blind was assumed if not reported.

#### Treatment length

Duration in weeks was extracted for each treatment arm.

#### Study quality

The Quality Assessment Tool for Quantitative Studies [8, 9] was adopted to classify study quality into “weak”, “moderate”, or “strong”. Final categorization was based on selection bias, study design, confounders, blinding, data collection methods, and withdrawals.

#### Sample diagnosis

For samples required to meet specific diagnostic criteria, required diagnoses were extracted; otherwise, the most common diagnosis was extracted [10].

#### Sample substance use

To examine sample representativeness [11–13], studies were coded based on explicit exclusion of individuals with substance use, regardless of frequency or severity.

#### Study region

Geographic locations (i.e., Africa, Asia, Oceania, Europe, North America, and South America) were extracted when reported; otherwise, the location of the first author’s primary affiliation was referenced.

#### Medication class

Medications were classified based on the *Textbook of Psychopharmacology* [14]. Categories included: (1) antidepressants; (2) anxiolytics; (3) antipsychotics; (4) mood stabilizers; (5) insomnia treatment agents; (6) psychostimulants for attention-deficit/hyperactivity disorder (ADHD); (7) nonpsychostimulants for ADHD; and (8) substance use disorder treatment agents. Antidepressants were further categorized into: (1) tricyclics/tetracyclics; (2) selective serotonin reuptake inhibitors (SSRIs); (3) serotonin-norepinephrine reuptake inhibitors (SNRIs); (4) norepinephrine-dopamine reuptake inhibitor antidepressant (NDRIs); (5) multimodal serotonin antagonists; (6) noradrenergic-specific serotonin antidepressants (NaSSAs); (7) monoamine oxidase inhibitors (MAOIs); and (8) N-methyl-d-aspartate (NMDA) receptor antagonists. Anxiolytics were further categorized as benzodiazepines or nonbenzodiazepines. Antipsychotics were categorized as typical or atypical.

#### Dose

Codes specified whether doses were specific or flexible. For set doses, the exact dose amount was extracted. For flexible doses, the mid-point was used.

#### Control group type

Control groups were classified as either active, placebo, or no treatment/ waitlist. In studies which leveraged an “active” control group, whichever medication was hypothesized by the authors to be the most efficacious was considered the “active” treatment.

#### Potential conflict of Interest

If articles disclosed receiving funding from the pharmaceutical industry, or if any study authors disclosed financial ties with the pharmaceutical industry (including employment), we coded the articles as having a potential conflict of interest (COI).

### Data analysis

All analyses were conducted in R (version 4.0.4) [15] using “metafor” [16] and “esc” [17]. To aggregate direct evidence within each trial, a head-to-head, pairwise meta-analysis was conducted using a frequentist random effects model.(In addition to the traditional pairwise approach, data were also analyzed using a random-effects Bayesian network meta-analysis. Due to space limitations, details of these analyses can be found in Supplemental Materials. Of note, network meta-analytic findings were largely consistent with those obtained from the pairwise meta-analysis.) Our analyses leveraged a tree-based approach: first, to assess whether medications were more efficacious at preventing certain SITBs than others, effect sizes were meta-analyzed based on reported outcomes. Next, to determine whether certain medications are more efficacious than others at preventing SITBs, effect sizes were meta-analyzed based on medication class. Last, metaregressions and sensitivity analyses were conducted. As noted previously, binary and continuous outcomes were considered separately in analyses to ensure that effect sizes shared the same scale and meaning [18]. Odds ratios were used as the primary effect size for analyses of binary outcomes, and standardized mean differences were used as the primary effect size for analyses of continuous outcomes.

Metaregressions examined the influence of publication year/era, sample severity, sample age and age group, sample size, sample sex, sample race, sample diagnosis, sample substance use, study region, intervention target, treatment length, and control group type. To assess small-study effects, a metaregression with treatment effects regressed against study variance was conducted. Sensitivity analyses were executed by excluding low quality and/or not double-blind studies. Publication bias was examined using Classic and Orwin’s Fail-Safe *N*, Begg and Mazumdar Rank Correlation Test, Egger’s Regression Test, and Duval and Tweedie’s Trim and Fill Test. As an additional risk of bias assessment, we also analyzed the potential influence of COIs on treatment effects.

## Results

### Descriptive statistics

#### Papers across time

**Fig. 1:**
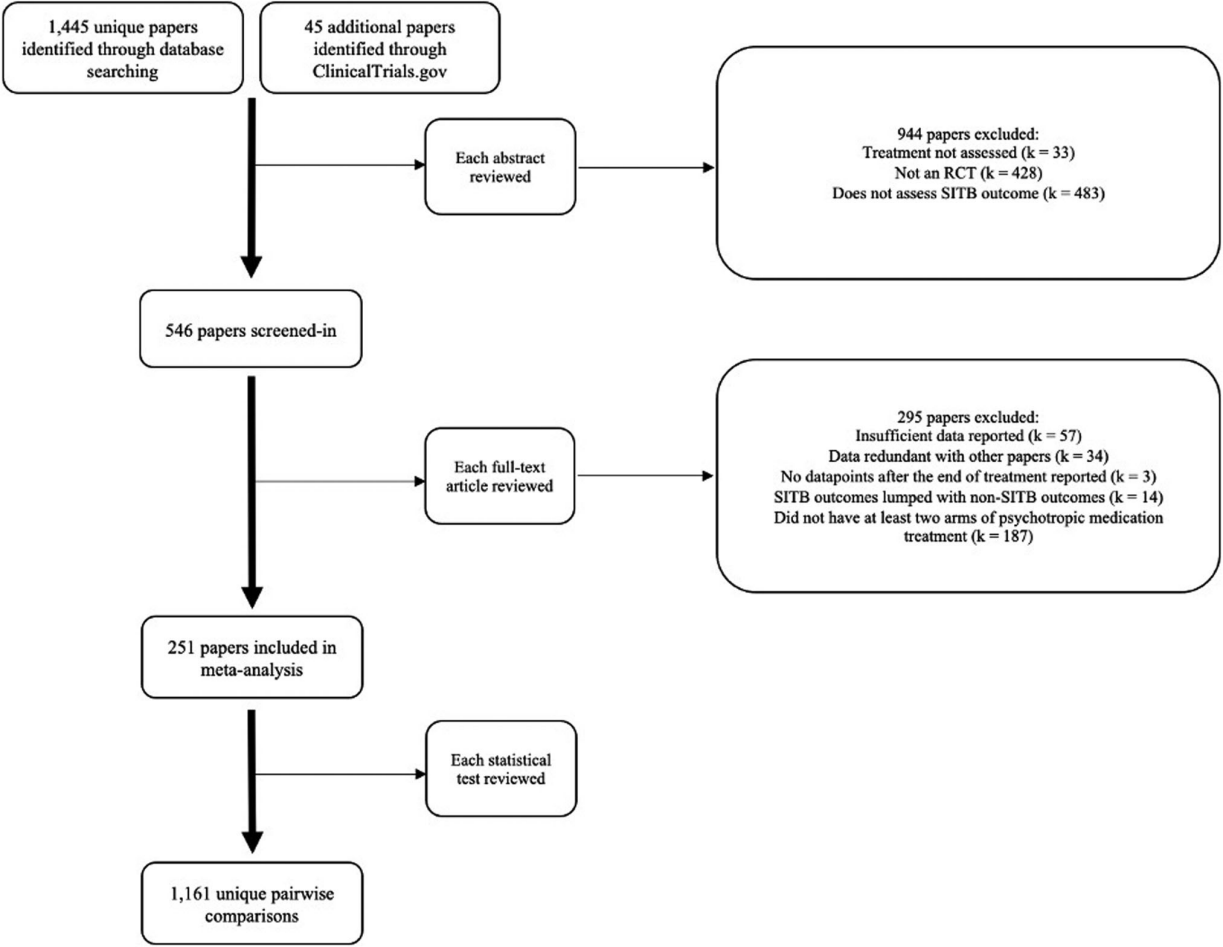
PRISMA Diagram. Flow diagram showing the study selection process.

Analyses included 251 papers comprising 718 RCTs and 1,161 pairwise comparisons (Fig. 1). Papers were published as early as 1964 [19], but most (59.36%) were published since 2010.

#### SITB outcomes

Most treatment effects (29.63%) focused on other/combined SITBs, followed by suicide ideation (27.99%), suicide death (19.04%), suicide attempt (16.11%), self-harm regardless of intent (4.39%), and NSSI (2.84%). Notably, 346 (29.80%) pairwise comparisons reported zero target events in both treatment arms. This was most common among psychostimulants for ADHD (65.00%), followed by insomnia treatment agents (54.55%), nonpsychostimulants for ADHD (45.83%), substance use disorder treatment agents (32.61%), antipsychotics (30.52%), mood stabilizers (28.26%), and antidepressants (24.24%). These effect sizes could not be meta-analyzed based on a frequentist approach due to insufficient variance to estimate treatment effects.

#### Sample severity

Almost all samples (92.83%) were clinical. The remainder were self-injurious (6.77%) or general (0.40%). Approximately half (53.64%) of clinical samples excluded participants on the basis of SITB risk. A small percentage of studies (3.98%) required included participants to exhibit SITBs prior to the start of the intervention. Most of these studies (80%) examined interventions designed to target SITBs; only two examined interventions targeting psychopathology broadly defined (i.e., depressive symptoms). Among these studies, primary outcomes of interest included suicide ideation (*n* = 2), suicide attempt (*n* = 8), suicide death (*n* = 5), self-harm regardless of intent (*n* = 2), and other/combined SITBs (*n* = 7). Examined medications included atypical antipsychotics (*n* = 7), typical antipsychotics (*n* = 2) mood stabilizers (*n* = 5), SSRIs (*n* = 4), tricyclic/tetracyclic antidepressants (*n* = 3), and substance use treatment agents (*n* = 3).

#### Sample age

Mean age was 33.15 (*SD* = 14.50). Most studies reporting age group focused on children/adolescents (30.06%), followed by adults (28.77%) and mixed older adults and adults (19.64%).

#### Sample sex

On average, females constituted 54.59% of the samples.

#### Sample race

Information regarding sample race was available for 54.78% of effects. On average, samples were 71.32% White/Caucasian, 18.33% Black, 9.45% Asian, 3.81% Indigenous, and 6.92% other (e.g., multiracial).

#### Sample diagnosis

Most studies reported sample diagnoses (95.61%). Most samples met criteria for depressive disorders (43.50%), followed by bipolar and related disorders (16.02%) and schizophrenia spectrum/other psychotic disorders (13.78%). No other diagnoses constituted more than 5% of the samples. Few studies recruited samples with a mix of different diagnoses (1.64%) or multiple simultaneous disorders (1.03%).

#### Sample substance use

Most comparisons (73.82%) were yielded from studies that excluded for substance use; 2.76% did not, and 23.43% did not specify.

#### Sample size

Papers pooling multiple RCTs reported sample sizes ranging from 268 to 48,277 (*median* = 2194). The remainder ranged from 18 to 18,154 (*median* = 363). Most sample sizes (63.39%) were below 500.

#### Study region

Most comparisons were drawn from studies conducted in North America (52.63%), and primarily the United States. Approximately 36.43% were from studies conducted across multiple continents, followed by Europe (5.00%), Asia (4.74%), Oceania (0.86%), South America (0.17%), and Africa (0.17%).

#### Intervention target

Most comparisons were drawn from interventions targeting psychopathology (93.54%), with a small percentage targeting SITBs (6.46%). Among interventions primarily targeting SITBs, approximately one-quarter of effects (28.00%) were drawn from studies which explicitly required SITBs for inclusion, whereas nearly half of effects (45.30%) were drawn from studies which excluded individuals on the basis of SITB risk.

#### Blind status

Most comparisons (94.06%) were from double-blind studies, followed by open-label (5.34%) and single-blind studies (0.60%).

#### Intervention components

Nearly all comparisons were drawn from interventions solely comprising psychotropic medication (97.24%). A small percentage (2.67%) of interventions combined medication with psychotherapy. One intervention (0.09%) combined medication with electroconvulsive therapy (ECT).

#### Control group type

The majority (61.70%) of interventions were compared to placebo, with the remainder (38.29%) compared to another active treatment. As active comparators varied widely across studies, an insufficient number of effect sizes were available to assess the effects of specific active comparators.

#### Treatment length

Treatments ranged from one day to three years (*median* = 8 weeks*, M* = 16.33, *SD* = 21.56).

#### Study quality

Most pairwise comparisons (58.74%) were associated with weak study quality [9], followed by moderate (35.83%) and strong (5.43%). No samples were rated “very likely” to be representative of the target population, most studies (78.29%) did not report what percentage of participants approached agreed to participate, and only 24.63% of comparisons were yielded from studies with retention rates above 80%.

#### Medication class

The most studied medications were antidepressants (50.82%), followed by antipsychotics (29.63%), psychostimulants for ADHD (5.17%), substance use disorder treatment agents (3.96%), mood stabilizers (3.96%), insomnia treatment agents (2.84%), nonpsychostimulants for ADHD (2.07%), and anxiolytics (0.60%). Very few studies examined a combination of medications (1.03%).

SSRIs were the most examined antidepressants (38.31%), followed by SNRIs (29.83%), multimodal serotonin antagonists (18.98%), NMDA receptor antagonists (5.42%), tricyclics/tetracyclics (3.90%), NaSSAs (1.19%), MAOIs (0.51%), and combinations of antidepressants (1.86%). Regarding anxiolytics, most comparisons examined benzodiazepines (71.43%) versus nonbenzodiazepines (28.57%). Among antipsychotics, most comparisons featured atypical (97.95%) versus typical antipsychotics (2.05%). Fluoxetine was the most studied medication (8.61%), followed by vilazodone (5.51%), duloxetine (5.08%), and quetiapine (5.08%; Table 1).

**Table 1:** Percentage of Medications Included in Pairwise Comparisons.

Medication	*n*	%	Dose	Medication	*n*	%	Dose
Fluoxetine	100	8.61%	31.00 (11.42)	Sertindole	9	0.78%	14.67 (3.16)
Vilazodone	64	5.51%	29.64 (9.72)	Viloxatine	9	0.78%	150.00 (54.77)
Duloxetine	59	5.08%	67.86 (25.62)	Ziprasidone	9	0.78%	100.00 (0.00)
Quetiapine	59	5.08%	409.23 (220.47)	Clozapine	8	0.69%	283.68 (26.80)
Desvenlafaxine	56	4.82%	81.07 (80.09)	Imipramine	8	0.69%	218.75 (45.81)
Cariprazine	54	4.65%	2.58 (2.28)	Levomilnacipran	8	0.69%	66.67 (20.66)
Venlafaxine	46	3.96%	138 (31.04)	Atomoxetine	7	0.60%	62.14 (12.19)
Asenapine	41	3.53%	5.34 (2.92)	Brexpiprazole	7	0.60%	2.32 (0.55)
Olanzapine	38	3.27%	12.31 (4.48)	Mirtazapine	7	0.60%	32.50 (0.00)
Paroxetine	38	3.27%	32.50 (6.18)	Buprenorphine	6	0.52%	0.45 (0.00)
Vortioxetine	38	3.27%	12.61 (5.03)	Edivoxetine	6	0.52%	13.00 (1.55)
Risperidone	36	3.10%	8.62 (10.91)	Maprotiline	6	0.52%	56.25 (21.65)
Paliperidone	34	2.93%	17.15 (29.17)	Haloperidol	5	0.43%	12.11 (5.00)
Sertraline	33	2.84%	134.72 (76.52)	Amitriptyline	3	0.26%	92.50 (56.62)
Citalopram	31	2.67%	32.50 (8.96)	Divalproex	3	0.26%	662.00 (942.24)
Lisdexamfetamine	30	2.58%	59.17 (21.80)	Fluvoxamine	3	0.26%	100.00 (0.00)
Lithium	24	2.07%	0.81 (0.16)	Selegiline Transdermal Patch	3	0.26%	20.00 (0.00)
Aripiprazole	22	1.90%	12.55 (5.96)	Zolpidem	3	0.26%	9.38 (0.00)
Suvorexant	21	1.81%	25.71 (9.01)	Carbamazepine	2	0.17%	7.06 (1.09)
Esketamine	18	1.55%	20.75 (2.62)	Etifoxine	2	0.17%	150.00 (0.00)
Varenicline	18	1.55%	1.94 (0.16)	Fluphenazine	2	0.17%	0.42 (0.00)
Escitalopram	17	1.46%	14.58 (3.34)	Lorazepam	2	0.17%	1.00 (0.00)
Lurasidone	16	1.38%	70.00 (25.98)	Memantine	2	0.17%	12.50 (0.00)
Ketamine	14	1.21%	0.53 (0.22)	Acamprosate	1	0.09%	1665.00 (−)
Guanfacine	13	1.12%	3.81 (0.26)	Chlordiazepoxide	1	0.09%	30.00 (−)
Lamotrigine	12	1.03%	243.75 (11.57)	Diazepam	1	0.09%	6.50 (−)
Armodafinil	11	0.95%	161.11 (22.05)	Mianserin	1	0.09%	30.00 (−)
Methylphenidate	11	0.95%	43.11 (6.17)	Modafinil	1	0.09%	200.00 (−)
Nalmefene	11	0.95%	16.67 (3.87)	Prazepam	1	0.09%	45.00 (−)
Bupropion	10	0.86%	261.11 (51.70)	Reboxetine	1	0.09%	6.00 (−)
Nefazodone	10	0.86%	337.50 (14.43)	Topiramate	1	0.09%	150.00 (−)
Eszopiclone	9	0.78%	2.00 (0.55)				

*Note*. *n* = number of pairwise comparisons with the specific medication as the active treatment; the dose unit is mg/day unless otherwise specified; the dose unit for ketamine is mg/kg; the dose unit for lithium is mEq/L; standard deviations could not be obtained for medications with only one pairwise comparison.

Bolded values indicate statistical significance at *p* < 0.05.

#### Dose

Most comparisons involved flexible doses (52.37%) versus fixed (40.05%; Table 1); 7.58% did not specify.

#### Potential COI

Most effect sizes (87.86%) were drawn from studies which disclosed financial ties to the pharmaceutical industry.

### Meta-analytic findings and publication bias

#### Overall effects

Analyses on binary outcomes included 759 effect sizes. On average, medications reduced binary SITB outcomes by approximately 8% (Table 2). Between-study heterogeneity was low (*I*^2^ = 14.92%). Analyses on continuous outcomes (*n* = 52) yielded a similarly small effect (Table 3); between-study heterogeneity was high (*I*^2^ = 91.98%). For binary outcomes, possible publication bias was detected via Egger’s test (*p* = .005), but other indices of publication bias were nonsignificant (Table 4). Significant evidence of publication bias was not detected for continuous outcomes (Table 4).

**Table 2:** Pairwise Meta-Analyses for Binary Outcomes.

	Overall		Suicide ideation		Suicide attempt		Suicide death	
	*n*	OR [95% CI]	*n*	OR [95% CI]	*n*	OR [95% CI]	*n*	OR [95% CI]
Psychotropic medications	**759**	**0.92 [0.87, 0.97]**	**268**	**0.85 [0.81, 0.98]**	**110**	**1.25 [1.01, 1.57]**	103	0.92 [0.77, 1.10]
Antidepressants	425	0.97 [0.90, 1.04]	134	0.91 [0.82, 1.00]	**68**	**1.36 [1.02, 1.82]**	49	1.19 [0.89, 1.60]
SSRIs	175	0.97 [0.86, 1.10]	34	0.88 [0.71, 1.09]	26	1.30 [0.84, 2.02]	22	0.92 [0.66, 1.27]
Citalopram	**19**	**0.63 [0.48, 0.83]**	**5**	**0.55 [0.41, 0.71]**	2	—	2	—
Escitalopram	14	1.14 [0.73, 1.76]	3	0.66 [0.13, 3.34]	2	—	3	1.82 [0.31, 10.54]
Fluoxetine	75	0.88 [0.76, 1.02]	13	1.02 [0.78, 1.32]	13	1.12 [0.66, 1.88]	2	—
Paroxetine	**32**	**2.19 [1.50, 3.21]**	**4**	**3.26 [1.17, 9.04]**			5	1.13 [0.44, 2.88]
Sertraline	28	1.02 [0.81, 1.27]	9	0.72 [0.47, 1.13]	4	1.49 [0.85. 2.61]	5	1.62 [0.55, 4.78]
SNRIs	121	0.98 [0.85, 1.12]	48	0.95 [0.80, 1.12]	17	1.02 [0.55, 1.87]	11	1.07 [0.42, 2.73]
Desvenlafaxine	35	0.89 [0.69, 1.15]	15	0.88 [0.65, 1.20]	7	0.88 [0.29, 2.63]	2	—
Duloxetine	41	1.01 [0.82, 1.24]	12	1.13 [0.86, 1.48]	6	0.39 [0.11, 1.42]	3	1.00 [0.17, 5.82]
Venlafaxine	33	1.11 [0.80, 1.54]	12	0.90 [0.59 1.38]	4	1.75 [0.73, 4.23]	5	1.00 [0.25, 3.99]
Multimodal serotonin antagonists	76	0.93 [0.82, 1.04]	39	0.90 [0.79, 1.03]	16	1.39 [0.77, 2.53]	1	—
Nefazodone	4	2.18 [0.64, 7.37]	0	—	2	—	1	—
Vilazodone	49	0.89 [0.78, 1.01]	31	0.89 [0.78, 1.01]	10	1.53 [0.60, 3.88]	0	—
Vortioxetine	23	1.13 [0.82, 1.57]	8	1.70 [0.71, 4.11]	0	—	0	—
NMDA receptor antagonists	19	0.79 [0.60, 1.04]	11	0.76 [0.52, 1.10]	1	—	0	—
Esketamine	13	0.86 [0.64, 1.17]	7	0.83 [0.55, 1.25]	1	—	0	—
Ketamine	6	0.46 [0.33, 4.90]	4	0.53 [0.23, 1.22]	0	—	0	—
Tricyclics and tetracyclics	17	1.00 [0.78, 1.29]	1	—	5	1.24 [0.55, 2.79]	7	1.39 [0.54, 3.58]
NaSSAs	7	1.88 [0.68, 5.22]	0	—	2	—	2	—
MAOIs	2	—	0	—	1	—	0	—
Antipsychotics	**213**	**0.80 [0.75, 0.87]**	73	0.91 [0.81, 1.03]	29	1.07 [0.73, 1.59]	42	0.79 [0.61, 1.01]
Atypical antipsychotics	**209**	**0.80 [0.74, 0.86]**	73	0.91 [0.81, 1.03]	27	0.32 [0.01, 8.21]	40	0.77 [0.59, 1.00]
Aripiprazole	11	0.55 [0.28, 1.10]	3	0.96 [0.37, 2.47]	1	—	3	0.50 [0.10, 2.47]
Asenapine	20	1.31 [0.89, 1.95]	12	1.35 [0.89, 2.05]	1	—	2	—
Cariprazine	29	0.90 [0.70, 1.17]	23	0.91 [0.70, 1.19]	2	—	2	—
Lurasidone	5	1.11 [0.81, 1.52]	4	1.14 [0.82, 1.59]	0	—	0	—
Olanzapine	17	0.80 [0.48, 1.34]	2	—	4	0.43 [0.09, 2.02]	8	0.92 [0.38, 2.23]
Paliperidone	**21**	**0.58 [0.36, 0.95]**	7	0.63 [0.35, 1.13]	2	—	5	0.39 [0.39, 1.64]
Quetiapine	43	0.95 [0.73, 1.22]	12	0.98 [0.59, 1.62]	4	1.29 [0.28, 6.01]	7	1.01 [0.31, 3.25]
Risperidone	32	1.01 [0.70, 1.45]	4	0.69 [0.42, 1.12]	**5**	**2.81 [1.53, 5.19]**	6	0.98 [0.33, 2.95]
Typical antipsychotics	**4**	**5.44 [1.47, 20.00]**	0	—	2	—	2	—
Psychostimulants for ADHD and narcolepsy	**21**	**1.77 [1.06, 2.94]**	15	1.63 [0.86, 3.09]	1	—	0	—
Armodafinil	**9**	**2.13 [1.02, 4.44]**	5	2.37 [0.85, 6.62]	0	—	0	—
Lisdexamfetamine	4	0.78 [0.17, 3.63]	3	0.52 [0.09, 3.01]	0	—	0	—
Agents for treatment of substance use disorders	27	0.81 [0.60, 1.10]	12	0.91 [0.65, 1.30]	2	—	3	0.84 [0.16, 4.54]
Bupropion	4	1.08 [0.66, 1.76]	2	—	0	—	1	—
Nalmefene	8	0.55 [0.28, 1.08]	3	0.76 [0.26, 2.20]	1	—	1	—
Varenicline	13	0.74 [0.45, 1.22]	7	0.78 [0.45, 1.35]			0	—
Mood stabilizers	29	0.88 [0.62, 1.22]	3	1.01 [0.52, 1.95]	8	1.15 [0.59, 2.27]	7	0.44 [0.13, 1.46]
Lamotrigine	11	1.87 [0.76, 4.57]	1	—	4	3.00 [0.60, 1.73]	2	—
Lithium	13	0.71 [0.47, 1.09]	1	—	3	0.80 [0.37, 1.73]	**5**	**0.23 [0.06, 0.97]**
Agents for treatment of insomnia	15	0.91 [0.55, 1.51]	13	0.88 [0.53, 1.49]	0	—	0	—
Suvorexant	9	1.57 [0.57, 4.34]	7	1.59 [0.51, 4.98]	0	—	0	—
Nonpsychostimulants for ADHD	13	0.84 [0.44, 1.65]	11	0.83 [0.40, 1.70]	0	—	0	—
Guanfacine	9	0.89 [0.41, 1.94]	7	0.89 [0.38, 2.06]	0	—	0	—
Anxiolytics	6	0.67 [0.18, 2.53]	1	—	1	—	2	—
Benzodiazepines	4	0.97 [0.19, 4.94]	0	—	1	—	2	—
Nonbenzodiazepines	2	—	1	—	0	—	0	—

	NSSI		Self-Harm		Other/Combined SITBs	
	n	OR [95% CI]	n	OR [95% CI]	n	OR [95% CI]
Psychotropic medications	**29**	**1.47 [1.04, 2.07]**	37	0.72 [0.47, 1.09]	**212**	**0.82 [0.77, 0.88]**
Antidepressants	**22**	**1.53 [1.07, 2.21]**	17	0.89 [0.44, 1.80]	**135**	**0.88 [0.81, 0.95]**
SSRIs	**7**	**1.90 [1.10, 3.28]**	6	0.94 [0.25, 3.50]	80	0.89 [0.76, 1.04]
Citalopram			0	—	10	0.85 [0.52, 1.40]
Escitalopram			2	—	4	1.21 [0.73, 2.00]
Fluoxetine	6	1.90 [0.79, 2.30]	1	—	**40**	**0.74 [0.63, 0.88]**
Paroxetine			3	1.40 [0.22, 8.97]	**15**	**2.42 [1.40, 4.20]**
Sertraline	1	—			9	1.02 [0.75, 1.38]
SNRIs	15	1.30 [0.80, 2.11]	7	0.82 [0.31, 2.17]	23	1.00 [0.74, 1.36]
Desvenlafaxine	4	0.63 [0.15, 2.62]	3	0.73 [0.21, 2.47]	4	1.00 [0.51, 1.92]
Duloxetine	9	1.36 [0.79, 2.31]	2	—	9	0.66 [0.42, 1.04]
Venlafaxine	2	—	2	—	8	1.45 [0.92, 2.29]
Multimodal serotonin antagonists	0	—	4	1.00 [0.20, 4.96]	16	0.93 [0.66, 1.31]
Nefazodone	0	—	0	—	1	—
Vilazodone	0	—	0	—	8	0.55 [0.23, 1.29]
Vortioxetine	0	—	4	1.00 [0.20, 4.96]	7	1.02 [0.70, 1.48]
NMDA receptor antagonists	0	—	0	—	7	0.83 [0.54, 1.27]
Esketamine	0	—	0	—	5	0.92 [0.57, 1.48]
Ketamine	0	—	0	—	2	—
Tricyclics and tetracyclics	0	—	0	—	4	0.96 [0.73, 1.26]
NaSSAs	0	—	0	—	0	—
MAOIs	0	—	0	—	1	—
Antipsychotics	3	0.34 [0.05, 2.16]	11	0.66 [0.30, 1.45]	**55**	**0.73 [0.65, 0.82]**
Atypical antipsychotics	3	0.34 [0.05, 2.16]	11	0.66 [0.30, 1.45]	**55**	**0.73 [0.65, 0.82]**
Aripiprazole	0	—	3	0.30 [0.08, 1.06]	1	—
Asenapine	0	—	0	—	3	0.33 [0.05, 2.12]
Cariprazine	0	—	0	—	2	—
Lurasidone	0	—	0	—	1	—
Olanzapine	0	—	1	—	2	—
Paliperidone	3	0.34 [0.05, 2.16]	3	1.55 [0.24, 9.99]	1	—
Quetiapine	0	—	3	1.01 [0.17, 5.89]	17	0.91 [0.67, 1.25]
Risperidone	0	—	0	—	17	0.75 [0.42, 1.32]
Typical antipsychotics	0	—	0	—	0	—
Psychostimulants for ADHD and narcolepsy	2	—	0	—	3	1.44 [0.38, 5.45]
Armodafinil	0	—	0	—	3	1.44 [0.38, 5.45]
Lisdexamfetamine	1	—	0	—	0	—
Agents for treatment of substance use disorders	0	—	2	—	**8**	**0.42 [0.19, 0.94]**
Bupropion	0	—	0	—	1	—
Nalmefene	0	—	1	—	3	0.45 [0.17, 1.16]
Varenicline	0	—	1	—	4	0.46 [0.09, 2.21]
Mood stabilizers	0	—	4	0.78 [0.33, 1.84]	7	0.76 [0.40, 1.45]
Lamotrigine	0	—	1	—	3	1.13 [0.26, 4.98]
Lithium	0	—	1	—	3	0.72 [0.33, 1.58]
Agents for treatment of insomnia	0	—	0	—	2	—
Suvorexant	0	—	0	—	2	—
Nonpsychostimulants for ADHD	2	—	0	—	0	—
Guanfacine	2	—	0	—	0	—
Anxiolytics	0	—	1	—	1	—
Benzodiazepines	0	—	0	—	1	—
Nonbenzodiazepines	0	—	1	—	0	—

Bolded values indicate statistical significance at *p* < 0.05.

**Table 3:** Pairwise meta-analyses for aggregated continuous outcomes.

Medications	All SITB Outcomes	
	n	*g* [95% CI]
Psychotropic medications	**52**	−**0.21 [**−**0.35,** −**0.06]**
Antidepressants	**21**	−**0.13 [**−**0.22,** −**0.05]**
SSRIs	10	−0.13 [−0.26, 0.01]
Citalopram	**3**	−**0.20 [**−**0.37,** −**0.04]**
Escitalopram	—	—
Fluoxetine	5	0.00 [−0.44, 0.45]
Paroxetine	—	—
Sertraline	—	—
SNRIs	—	—
Desvenlafaxine	—	—
Duloxetine	—	—
Venlafaxine	—	—
Multimodal serotonin antagonists	—	—
Nefazodone	—	—
Vilazodone	—	—
Vortioxetine	—	—
NMDA receptor antagonists	4	−0.05 [−0.44, 0.33]
Esketamine	—	—
Ketamine	4	−0.05 [−0.44, 0.33]
Tricyclics and tetracyclics	4	−0.10 [−0.28, 0.07]
NaSSAs	—	—
MAOIs	—	—
Antipsychotics	**24**	−**0.27 [**−**0.53,** −**0.01]**
Atypical antipsychotics	21	−0.20 [−0.41, 0.01]
Aripiprazole	6	0.05 [−0.08, 0.17]
Asenapine	—	—
Cariprazine	—	—
Lurasidone	3	−0.10 [−0.22, 0.03]
Olanzapine	5	−0.57 [−1.28, 0.13]
Paliperidone	3	−0.03 [−0.14, 0.09]
Quetiapine	—	—
Risperidone	—	—
Typical antipsychotics	—	—
Psychostimulants for ADHD	—	—
Armodafinil	—	—
Lisdexamfetamine	—	—
Agents for treatment of substance use disorders	**4**	−**0.38 [**−**0.70,** −**0.05]**
Bupropion	—	—
Nalmefene	—	—
Varenicline	—	—
Mood stabilizers	3	−0.19 [−1.09, 0.71]
Lamotrigine	—	—
Lithium	—	—
Agents for treatment of insomnia	—	—
Suvorexant	—	—
Nonpsychostimulants for ADHD	—	—
Guanfacine	—	—
Anxiolytics	—	—
Benzodiazepines	—	—
Nonbenzodiazepines	—	—

Bolded values indicate statistical significance at *p* < 0.05.

**Table 4:** Publication Bias.

	Fail-Safe *N*		Begg and Mazumdar Rank correlation	Egger’s Test of Intercept	Duval and Tweedie’s Trim and Fill	
Binary/Categorical	Classic	Orwin’s			Missing effect sizes	Adjusted OR
Overall	551	0	τ = −0.02, *p* = 0.49	z = 2.80, *p* = 0.005	0	—
Suicide Ideation	177	131	τ = −0.03, *p* = 0.50	z = 0.69, *p* = 0.49	0	—
Suicide Attempt	109	0	τ = 0.004, *p* = 0.95	z = 1.96, *p* = 0.05	0	—
Suicide Death	0	79	τ = −0.14, *p* = 0.04	z = 0.46, *p* = 0.64	1	0.93 [0.78, 1.11]
NSSI	4	0	τ = −0.13, *p* = 0.34	z = −0.66, *p* = 0.51	1	1.52 [1.08, 2.15]
Self-Harm	0	161	τ = 0.14, *p* = 0.21	z = 0.47, *p* = 0.64	0	—
Other/Combined SITBs	558	276	τ = −0.03, *p* = 0.52	z = 1.50, *p* = 0.13	0	—
	**Fail-Safe** ***N***		**Begg and Mazumdar Rank correlation**	**Egger’s Test of Intercept**	**Duval and Tweedie’sTrim and Fill**	
**Continuous**	**Classic**	**Orwin’s**			**Missing effect sizes**	**Adjusted** ***g***
Overall	1808	151	τ = −0.21, *p* = 0.03	z = 0.76, *p* = 0.45	0	—
Suicide Ideation	0	0	τ = 0.07, *p* = 0.90	z = 0.68, *p* = 0.50	0	—
Suicide Attempt	—	—	—	—	—	—
Suicide Death	—	—	—	—	—	—
NSSI	—	—	—	—	—	—
Self-Harm	—	—	—	—	—	—
Other/Combined SITBs	—	—	—	—	—	—

Bolded values indicate statistical significance at *p* < 0.05.

Antipsychotics yielded approximately a 20% reduction in binary outcomes; no other medication classes appeared efficacious (Table 2). Regarding specific medications, citalopram and paliperidone on average reduced SITBs by approximately 40%, whereas armodafinil and paroxetine appeared to increase risk (Table 2). Analyses of continuous outcomes indicated significant treatment effects for antidepressants, antipsychotics, substance use disorder treatment agents, and citalopram (Table 3).

#### Suicide ideation

Medications on average, but no specific classes, slightly reduced suicide ideation. Citalopram was the only specific medication that reduced suicide ideation (by approximately 45%); paroxetine increased its likelihood (Table 2). Significant evidence of publication bias was not detected (Table 4).

#### Suicide attempt

Medications on average were associated with modestly increased odds of suicide attempt. No specific classes or medications appeared efficacious for preventing suicide attempts. Antidepressants and risperidone appeared to increase risk (Table 2). Significant evidence of publication bias was not detected (Table 4).

#### Suicide death

Overall, medications did not influence suicide death. Only lithium significantly reduced risk, by approximately 77% (Table 2). Possible publication bias was detected by Duval and Tweedie’s trim and fill test; however, adjusted effects differed minimally from detected effects (Table 4).

#### NSSI

Overall, medications increased the odds of NSSI; however, when analyzed by class, these effects were only observed for antidepressants (Table 2). Possible publication bias was detected by Duval and Tweedie’s trim and fill test; however, adjusted effects differed minimally from detected effects (Table 4).

#### Self-harm

No significant treatment effects for any medications or classes were detected for self-harm (Table 2). Significant evidence of publication bias was not detected (Table 4).

#### Other/combined SITBs

Overall, medications reduced other/combined SITBs by approximately 18% (Table 2). Antidepressants (and fluoxetine specifically), atypical antipsychotics, and substance use disorder treatment agents produced significant treatment effects. Paroxetine increased the odds of other/combined SITBs (Table 2). Significant evidence of publication bias was not detected (Table 4).

### Moderator analyses

#### Publication year and era

When publication year was considered a continuous outcome, it did not appear to moderate effect estimates (Table 5). When comparisons were categorized into publication era, effect estimates obtained from studies published in the 2000s were significantly weaker compared with those from the 1960s and 1970s. For suicide death, effect estimates obtained from studies published in the 1990s were found to be significantly weaker than those from the 1960s and 1970s.

**Table 5:** Moderator analyses for pairwise meta-analyses.

Moderators	Overall		Suicide Ideation		Suicide Attempt		Suicide Death	
	b	95% CI	b	95% CI	b	95% CI	b	95% CI
Publication year	0.01	[−0.00, 0.01]	0.01	[−0.00, 0.03]	−0.01	[−0.04, 0.02]	−0.02	[−0.05, 0.01]
Publication era								
1960s–1970s	Reference		—	—	—	—	Reference	
1980s	−1.56	[0.27, 1.16]	—	—	Reference		—	—
1990s	1.06	[−0.40, 2.51]	Reference		−0.42	[−1.92, 1.59]	**1.81**	**[0.01, 3.60]**
2000s	**1.46**	**[0.02, 2.91]**	−0.23	[−1.16, 0.70]	0.10	[−2.24, 1.44]	1.52	[−0.08, 3.12]
2010s	1.25	[−0.20, 2.70]	−0.14	[−1.03, 0.76]	−0.22	[−1.58, 1.14]	1.05	[−0.55, 2.65]
2020 s	1.29	[−0.20, 2.70]	0.13	[−0.87, 1.12]	−0.29	[−2.58, 2.00]	0.31	[−2.46, 3.08]
Sample severity								
General	Reference		Reference		Reference		Reference	
Clinical (SITB-excluded)	−0.10	[−0.59, 0.39]	0.20	[−0.43, 0.83]	−0.08	[−1.22, 1.38]	−0.56	[−1.29, 0.16]
Clinical (not SITB-excluded)	−0.06	[−0.55, 0.43]	0.16	[−0.47, 0.79]	−0.22	[−1.49, 1.05]	—	—
SITB	−0.35	[−0.87, 0.16]	−0.00	[−0.73, 0.72]	−0.84	[−2.21, 0.52]	−0.22	[−2.14, 1.70]
Sample age	−**0.01**	**[**−**0.01,** −**0.00]**	−**0.01**	**[**−**0.01,** −**0.00]**	−0.02	[−0.04, 0.00]	0.04	[−0.00, 0.08]
Sample age group								
Adults only	Reference		Reference		Reference		Reference	
All age groups	−0.12	[−0.33, 0.09]	—	—	—	—	—	—
Children/adolescents only	**0.39**	**[0.25, 0.54]**	**0.25**	**[0.06, 0.44]**	0.41	[−0.28, 1.09]	—	—
Mixed adolescents and adults	0.29	[−0.03, 0.61]	0.27	[−0.15, 0.69]	−0.11	[−1.91, 1.69]	−0.26	[−1.88, 1.37]
Mixed older adults and adults	0.09	[−0.04, 0.24]	0.11	[−0.06, 0.28]	0.11	[−0.55, 0.76]	0.24	[−0.38, 0.86]
Older adults only	−0.22	[−0.87, 0.44]	−0.28	[−1.02, 0.46]	−1.64	[−4.89, 1.62]	0.91	[−1.02, 2.84]
Did not report	0.15	[−0.01, 0.31]	−0.14	[−0.40, 0.13]	0.42	[−0.13, 0.97]	**0.75**	**[0.17, 1.34]**
Sample sex	**0.01**	**[0.01, 0.01]**	0.01	[−0.00, 0.01]	0.01	[0.00, 0.03]	0.02	[−0.00, 0.04]
Sample race (% white)	0.002	[−0.003, 0.01]	0.003	[−0.003, 0.01]	0.01	[−0.01, 0.03]	0.001	[−0.02, 0.03]
Sample diagnosis								
Anxiety disorders	−0.04	[−0.11, 0.02]	−0.07	[−0.39, 0.25]	—	—	—	—
Bipolar and related disorders	−0.07	[−0.38, 0.24]	0.13	[−0.07, 0.32]	0.20	[−0.59, 0.99]	−0.52	[−1.39, 0.36]
Conditions for further study	−0.05	[−0.13, 0.21]	−0.00	[−0.78, 0.78]	—	—	—	—
Depressive disorders	Reference		Reference		Reference		Reference	
Feeding and eating disorders	0.02	[−1.75, 1.80]	−0.41	[−2.52, 1.71]	—	—	—	—
Neurodevelopmental disorders	−0.14	[−0.62, 0.33]	−0.11	[−0.62, 0.40]	—	—	—	—
Obsessive-compulsive disorders	**−0.53**	**[−0.81, −0.24]**	1.26	[−1.96, 4.49]	—	—	—	—
Personality disorders	−0.53	[−1.11, 0.06]	—	—	−**1.04**	**[−2.00, −0.07]**	−1.32	[−4.57, 1.93]
Schizophrenia spectrum and other psychotic disorders	**−0.27**	**[−0.41, −0.14]**	**−0.28**	**[−0.56, −0.00]**	−0.24	[−0.76, 0.28]	−0.41	[−0.88, 0.07]
Sleep-wake disorders	0.15	[−0.51, 0.82]	0.17	[−0.52, 0.85]	—	—	—	—
Substance-related and addictive disorders	−0.34	[−0.77, 0.10]	−0.16	[−0.70, 0.38]	—	—	−0.10	[−1.68, 1.49]
Trauma- and stressor-related disorders	−1.07	[−2.94, 0.80]	−1.03	[−3.32, 1.25]	—	—	—	—
Mixed diagnoses	−0.12	[−0.63, 0.38]	0.06	[−0.65, 0.77]	−0.91	[−2.59, 0.76]	−0.12	[−1.15, 0.91]
Multiple diagnoses	0.39	[−0.79, 1.57]	−0.08	[−1.60, 1.43]	0.11	[−3.30, 3.53]	—	—
Did not report	**0.47**	**[0.20, 0.74]**	—	—	—	—	−0.15	[−0.70, 0.40]
Sample substance use exclusion	0.08	[−0.11, 0.26]	−0.07	[−0.31, 0.17]	0.96	[−0.02, 1.94]	−0.42	[−1.90, 1.05]
Sample size	**−0.05**	**[−0.09, −0.01]**	**−0.08**	**[−0.16, −0.00]**	−0.10	[−0.23, 0.03]	−0.01	[−0.11, 0.08]
Study region								
Africa	−1.04	[−3.34, 1.26]	−0.95	[−4.18, 2.28]	—	—	—	—
Asia	0.02	[−0.23, 0.27]	0.15	[−0.10, 2.28]	−0.22	[−2.01, 1.57]	−0.69	[−1.86, 0.49]
Europe	−2.00	[−0.49, 0.10]	−0.38	[−0.89, 0.13]	−0.61	[−1.26, 0.03]	−0.12	[−0.89, 0.64]
Multiple sites across continents	−0.06	[−0.17, 0.06]	0.15	[0.00, 0.30]	**−0.70**	**[−1.19, −0.22]**	**−0.40**	**[−0.78, −0.02]**
North America	Reference		Reference		Reference		Reference	
Oceania	0.32	[−0.07, 0.71]	0.45	[−0.08, 0.98]	—	—	—	—
South America	−1.06	[−4.34, 2.21]	−0.98	[−4.23, 2.28]	—	—	—	—
Treatment length	**−0.11**	**[−0.16, −0.06]**	−0.00	[−0.07, 0.02]	**−0.28**	**[−0.47, −0.08]**	**−0.27**	**[−0.47, −0.08]**
Intervention target type								
Psychopathology	Reference		Reference		Reference		Reference	
SITBs	**−0.21**	**[−0.35, −0.06]**	0.04	[−0.23, 0.31]	**−0.85**	**[−1.40, −0.30]**	−0.37	[−0.86, 0.12]
Intervention components								
Medication only	Reference		Reference		Reference		Reference	
Medication and psychotherapy	0.23	[−0.06, 0.51]	0.17	[−0.26, 0.61]	0.43	[−1.13, 1.99]	−1.56	[−4.61, 1.49]
Control group type								
Active treatment	Reference		Reference		Reference		Reference	
Placebo	0.01	[−0.09, 0.12]	−0.12	[−0.26, 0.02]	**0.45**	**[0.01, 0.89]**	**0.54**	**[0.14, 0.94]**
Small-study effects	0.06	[−0.01, 0.13]	0.02	[−0.11, 0.14]	0.16	[−0.05, 0.36]	−0.01	[−0.19, 0.16]

Moderators	NSSI		Self-Harm		Other/Combined SITBs	
	b	95% CI	b	95% CI	b	95% CI
Publication year	0.11	[−0.01, 0.23]	0.07	[−0.03, 0.17]	0.01	[−0.00, 0.01]
Publication era						
1960s–1970s	—	—	—	—	Reference	
1980s	—	—	—	—	—	—
1990s	—	—	—	—	0.90	[−2.42, 4.22]
2000s	Reference		Reference		1.34	[−1.98, 4.66]
2010s	0.11	[−2.02, 2.24]	0.89	[−0.11, 1.90]	1.00	[−2.31, 4.32]
2020 s	—	—	—	—	0.80	[−2.57, 4.17]
Sample severity						
General	Reference		Reference		Reference	
Clinical (SITB-excluded)	—	—	—	—	−0.02	[−1.98, 1.95]
Clinical (not SITB-excluded)	—	—	—	—	0.13	[−1.83, 2.10]
SITB	—	—	—	—	−0.16	[−2.12, 1.81]
Sample age	0.06	[−0.03, 0.15]	−0.00	[−0.03, 0.03]	**−0.01**	**[−0.01, −0.00]**
Sample age group						
Adults only	Reference		Reference		Reference	
All age groups	—	—	—	—	0.05	[−0.13, 0.23]
Children/adolescents only	−0.89	[−4.12, 2.35]	0.29	[−0.94, 1.53]	**0.62**	**[0.40, 0.85]**
Mixed adolescents and adults	−0.22	[−3.50, 3.06]	−1.05	[−3.01, 0.92]	0.09	[−0.57, 0.75]
Mixed older adults and adults	−0.93	[−4.59, 2.73]	0.20	[−1.37, 1.78]	0.08	[−0.12, 0.28]
Older adults only	—	—	—	—	−0.40	[−2.46, 1.66]
Did not report	—	—	0.15	[−1.15, 1.45]	−0.27	[−0.58, 0.04]
Sample sex	−0.03	[−0.02, 0.07]	0.00	[−0.02, 0.03]	**0.01**	**[0.00, 0.02]**
Sample race (% white)	0.03	[−0.01, 0.06]	0.003	[−0.04, 0.05]	0.01	[−0.002, 0.01]
Sample diagnosis						
Anxiety disorders	0.03	[−1.60, 1.65]	−0.17	[−2.20, 1.78]	−0.11	[−0.81, 2.80]
Bipolar and related disorders	—	—	−0.21	[−1.35, 0.79]	1.00	[−0.60, 0.38]
Conditions for further study	—	—	—	—	—	—
Depressive disorders	Reference		Reference		Reference	
Feeding and eating disorders	0.66	[0.57, 3.90]	—	—	—	—
Neurodevelopmental disorders	−0.01	[−1.69, 1.66]	−0.48	[−3.03, 2.06]	−0.95	[−4.18, 2.28]
Obsessive-compulsive disorders	—	—	—	—	**−0.46**	**[−0.73, −0.19]**
Personality disorders	—	—	0.49	[−1.98, 0.99]	0.62	[−1.39, 2.62]
Schizophrenia spectrum and other psychotic disorders	−1.52	[−3.42, 0.38]	0.19	[−1.21, 1.59]	**−0.28**	**[−0.44, −0.13]**
Sleep-wake disorders	—	—	—	—	0.50	[−1.77, 2.77]
Substance-related and addictive disorders	—	—	−0.37	[−2.75, 2.02]	−0.69	[−1.51 0.13]
Trauma- and stressor-related disorders	—	—	−0.94	[−4.24, 2.35]	—	—
Mixed diagnoses	—	—	—	—	−0.59	[−2.08, 0.90]
Multiple diagnoses	—	—	—	—	1.54	[−0.68, 3.77]
Did not report	—	—	—	—	**0.70**	**[0.37, 1.02]**
Sample substance use exclusion	−0.66	[−1.45, 0.15]	−0.09	[−1.12, 0.95]	0.18	[0.00, 0.37]
Sample size	−0.40	[−0.99, 0.20]	0.05	[−0.47, 0.58]	−0.03	[−0.09, 0.02]
Study region						
Africa	—	—	−0.60	[−3.95, 2.75]	—	—
Asia	—	—	−0.52	[−1.34, 2.38]	−0.70	[−2.30, 0.90]
Europe	—	—	−0.66	[−2.42, 1.11]	0.10	[−0.60, 0.80]
Multiple sites across continents	−0.06	[−2.21, 2.08]	0.32	[−0.76, 1.41]	**−0.17**	**[−0.31, −0.03]**
North America	Reference		Reference		Reference	
Oceania	0.59	[−1.62, 2.80]	—	—	−0.23	[−0.91, 0.45]
South America	—	—	—	—	—	—
Treatment length	0.39	[−0.37, 1.15]	0.08	[−0.31, 0.46]	**−0.09**	**[−0.15, −0.03]**
Intervention target type						
Psychopathology	Reference		Reference		Reference	
SITBs	−0.63	[−1.38, 0.11]	—	—	**−0.16**	**[−0.30, −0.01]**
Intervention components						
Medication only	Reference		Reference		Reference	
Medication and psychotherapy	0.65	[−0.14, 1.45]	0.42	[−1.14, 1.98]	0.16	[−0.23, 0.55]
Control group type						
Active treatment	Reference		Reference		Reference	
Placebo	−0.15	[−0.85, 0.55]	0.15	[−0.71, 1.01]	0.04	[−0.10, 0.18]
Small-study effects	−0.10	[−0.54, 0.34]	0.13	[−0.34, 0.59]	0.07	[−0.06, 0.20]

Bolded values indicate statistical significance at *p* < 0.05.

#### Sample severity

Treatment effects did not significantly differ depending on whether participants were required to exhibit SITBs or psychopathology for study inclusion, or whether participants were excluded on the basis of SITB risk (Table 5). Further, no significant moderating effects of sample severity were detected for any specific SITB outcomes.

#### Sample age and age group

Overall, treatment effects appeared weaker among younger samples; these effects were also detected for suicide ideation and other/combined SITBs specifically (Table 5). In addition to studying sample age as a continuous variable, we also examined sample age as a categorical variable. When all outcomes were aggregated, samples with children and adolescents only were associated with significantly weaker treatment effects compared with samples with adults only; again, similar effects emerged for suicide ideation and other/combined SITBs (Table 5). For suicide death, studies that did not report sample age group were associated with significantly weaker effects (Table 5). No other significant effects were detected.

#### Sample sex

When all SITB outcomes were aggregated, samples with higher percentages of female participants yielded significantly weaker treatment effects (Table 5). This moderator effect was also detected for other/combined SITBs, but not for other specific outcomes (Table 5).

#### Sample race

No moderating effects were detected based on the percentage of the sample that was White (versus non-White) either when SITB outcomes were aggregated, or for any specific SITB outcomes (Table 5).

#### Sample diagnosis

Overall, treatment effects appeared stronger among samples with primary diagnoses of obsessive-compulsive disorders and schizophrenia spectrum and other psychotic disorders compared with samples with depressive disorders as their primary diagnosis (Table 5). Samples with unknown primary diagnoses were associated with significantly weaker treatment effects. These three moderator effects were also found for other/combined SITBs (Table 5). For suicide ideation, stronger treatment effects were found among samples with primary diagnoses of schizophrenia spectrum and other psychotic disorders; for suicide attempt, samples where participants met diagnostic criteria for personality disorders were associated with stronger treatment effects (Table 5). All other effects were nonsignificant.

#### Sample substance use

Whether studies excluded participants with substance use did not significantly moderate treatment effects when all SITB outcomes were considered together or for specific SITB outcomes (Table 5).

#### Sample size

For aggregated SITBs, larger sample sizes were associated with significantly stronger treatment effects (Table 5). Sample size also significantly moderated treatment effects for suicide ideation, but not for other outcomes (Table 5).

#### Study region

When all SITBs were considered together, the region where the studies were conducted did not significantly moderate treatment effects (Table 5). Studies conducted at multiple sites across different continents, however, were associated with stronger treatment effects for suicide attempt and other/combined SITBs (Table 5).

#### Treatment length

Longer treatment duration was associated with stronger treatment effects overall (Table 5). Similar effects were found for suicide attempt, suicide death, and other/combined SITBs (Table 5).

#### Intervention target type

Interventions that intended to primarily target SITBs yielded significantly stronger treatment effects when all the outcomes were pooled together (Table 5), as well as for suicide attempt and other/combined SITBs.

#### Intervention components

Interventions that included additional components (e.g., psychotherapy) did not produce significantly different treatment effects than interventions which solely involved medication when all SITB outcomes were considered together or for specific SITB outcomes (Table 5).

#### Control group type

When all SITBs were considered together, control group type (i.e., active or placebo) did not significantly moderate treatment effects (Table 5). However, significant effects were detected for suicide attempt and suicide death, such that interventions compared to placebo yielded weaker treatment effects than interventions compared to other active treatments (Table 5). No significant moderating effects of control group type were detected when medication classes were considered separately (Supplemental Materials).

### Small-study effects

No small-study effects were significant (Table 5).

### Sensitivity analyses

Analyses excluding studies that did not have a double-blind design or were rated as “weak” quality were statistically consistent with analyses including those studies.

### COI risk of bias

Metaregressions revealed a significant moderating effect of potential COIs (b = −0.21, *p* = 0.02), such that articles disclosing financial ties to the pharmaceutical industry reported significantly stronger treatment effects than articles which did not. Studies disclosing potential COIs were more likely to report weak yet beneficial effects of treatment, whereas studies without COIs were more likely to report potentially iatrogenic effects of treatment (RR’s 0.90 [0.87, 0.94] vs. 1.16 [0.93, 1.44], respectively).

## Discussion

Despite an increase in psychotropic medication prescription over the years [20–25], SITB rates have not decreased [1, 2]. It is critical to establish whether psychotropic medications represent an efficacious treatment for SITBs, yet firm evidence regarding the efficacy of medications for treating and preventing SITBs has remained elusive. While prior narrower reviews and meta-analyses in this domain have provided valuable insights into the effects of specific medications on particular SITBs within certain populations [3–5], the present study aimed to substantially advance scientific understanding in this domain by summarizing all published RCTs reporting the causal effects of psychotropic medications on SITBs. In addition to evaluating the overall efficacy of psychotropic medications on SITBs, we aimed to determine which medications were most efficacious for which specific types of SITBs, and whether psychotropic medication efficacy was influenced by a variety of study and sample characteristics.

The present investigation yielded several main findings. Psychotropic medications produced, on average, an 8% reduction in SITB occurrence and a reduction of 0.2 standard deviations in SITB symptoms and severity. Although effects differed by medication and medication class, most medications examined in the present study did not significantly impact SITBs. Only two specific medications (i.e., citalopram, ketamine) and one medication class (i.e., antipsychotics) produced larger-than-average treatment effects. Because the most efficacious medications tended to be studied less frequently (and in the case of ketamine, over a much shorter treatment interval), additional evidence is needed to corroborate the potential superiority of these medications. If evidence of their efficacy is corroborated, future research is also needed to understand their treatment mechanisms. Conversely, two medications (i.e., psychostimulants, typical antipsychotics) were found to increase SITB risk. Because we intended to examine the treatment effects of psychotropic medications on SITBs rather than iatrogenic effects, these findings should be interpreted with caution; additional research is needed to corroborate the potential iatrogenic effects detected in this study. If iatrogenic effects are substantiated [26, 27], these interventions are unlikely to be appropriate for treating SITBs.

Efficacy also differed by outcome. Medications overall were efficacious for suicide ideation and other/combined SITBs, though effects were small; however, they appeared to increase the odds of suicide attempt and NSSI, and they did not significantly impact suicide death and self-harm. Different SITBs may have distinct neurobiological pathways, and certain medications may impact some pathways but not others; future research is needed to test this possibility.

Some moderators (i.e., sample age, sample diagnosis, treatment length, treatment target, geographic location) influenced treatment effects slightly. Moderator effects were not consistent across outcomes, however, suggesting that treatment effects are related to study and sample characteristics, but not robustly. Nevertheless, it is notable that nearly half of included studies excluded individuals on the basis of SITB risk, and a very small number required SITBs for inclusion. This is likely to limit the generalizability of findings. Future medication trials should prioritize including individuals experiencing SITBs. Similarly, because only a small number of studies (k = 17) evaluated interventions targeting SITB specifically, future RCTs would benefit from continuing to evaluate whether psychotropic medications are efficacious for addressing SITBs when they are prescribed for that purpose. Additional studies are necessary to further clarify the effects of using psychotropic medications to address SITBs as a primary treatment aim.

Within the constraints of the existing literature, findings suggest that only a small proportion of psychotropic medications are efficacious for a few specific SITBs. Importantly, averaged effects do not guarantee any individual’s response, and the present meta-analysis cannot provide insights into potential idiographic effects of medications. As suggested by precision medicine approaches and personalized models of psychopathology, certain interventions may only be consistently and significantly efficacious for a subset of populations. In other words, it is possible that a large effect for a small subset of patients may be present despite a null effect for most patients. We cannot rule out this possibility, as the present meta-analysis can only speak to nomothetic medication effects. In addition to efficacy, toxicity, tolerability, side effects, interactions, and ease of administration should also be considered when prescribing medications. Different interventions may be preferred for certain clinical situations.

Our findings are consistent with previous smaller-in-scope reviews that either found no significant effects of psychotropic medications on SITBs, or only found preliminary evidence for potential efficacy [28–31]. The present study represents an extension of a broad meta-analysis which found that the aggregated effects of psychotropic medications on SITBs were small [6]. We hoped that by providing a more intensive investigation of the efficacy of psychotropic medications for SITBs, and by examining whether efficacy differed across SITB outcomes, specific medications, or different populations, we might detect findings that were obscured in the larger meta-analysis. Instead, we found that although a small number of psychotropic medications were efficacious for a few specific SITB outcomes, most psychotropic medications do not appear to exert significant effects on SITBs. These effects were largely consistent regardless of medication type, SITB outcome type, and sample and study characteristics. However, it is important to consider these findings in light of the limitations of the existing literature, which are delineated below.

### Limitations

Meta-analytic results reported herein are influenced by the methodological constraints of the primary studies that qualified for inclusion. First, certain medications may be efficacious, but have yet to be adequately tested within RCTs [32, 33]. Second, many trials administered approximately half the maximum dose allowed by the Food and Drug Administration; certain medications may be more efficacious at higher doses. Third, the majority of analyzed studies disclosed financial ties to the pharmaceutical industry, which may have biased findings. Supporting this possibility, we found that studies with potential COIs were likely to report stronger treatment effects.

Fourth, most studies examined SITBs as discrete, rather than continuous, outcomes. As medications may be more efficacious at reducing SITB severity than preventing SITBs, future studies are encouraged to prioritize capturing effects on severity. Fifth, in studies where SITBs were not considered the primary treatment target, they may not have been measured in a way that would accurately reflect medication effects. Future RCTs should aim to intentionally examine the effects of psychotropic medications on SITBs.

Sixth, most studies excluded participants with a history of substance use. Whereas some studies only excluded participants who met the criteria for a substance use disorder, several excluded participants simply on the basis of recent illicit substance use (e.g., marijuana use within the last 30 days). Substance use is often present among populations seeking psychiatric care [11, 12], and substance use disorders may be comorbid with diagnoses commonly associated with SITBs [13, 34]. Thus, the effect estimates observed in the present study may not apply to real-world clinical settings. In a similar vein, fewer than 2% of comparisons were yielded from samples with primary personality disorder diagnoses. Personality disorders are often associated with more chronic and severe SITBs [35, 36]. The small number of studies on this population represents a major gap in knowledge. Taken together, RCT samples may not be representative of patients seeking or receiving medications for SITBs.

Seventh, most RCTs targeted mental disorders instead of SITBs. Consequently, studies may have prioritized recruiting samples meeting certain diagnostic criteria rather than meeting a SITB severity threshold. Indeed, a very small percentage of included studies (3.98%) specifically required individuals to exhibit SITBs, and nearly half of analyzed effects (49.87%) were drawn from studies that *excluded* individuals on the basis of SITB risk. This may limit the generalizability of our findings to those at highest risk of SITBs. It is critical that future RCTs intentionally include individuals with SITBs to further clarify treatment effects in self-injurious and suicidal populations. This is especially critical for RCTs evaluating interventions intended to address SITBs as a primary treatment aim.

Eighth and finally, examined medications were generally not developed to target the pharmacological origins of SITBs [37]; instead, many were discovered serendipitously without a clear understanding of their mechanisms. Rather than attempting to draw causal inferences based on treatment response, future research should prioritize understanding the pharmacological causes of SITBs *before* developing corresponding medications to expedite intervention development and improvement.

This study also has methodological limitations. First, we could not elucidate idiographic effects. Certain medications may only be reliably efficacious for some, which would also result in an overall small effect. Second, as with most meta-analyses, it is possible that not all qualifying studies were included. This study is an extension of a prior meta-analysis evaluating the efficacy of a wide variety of SITB interventions. Given the scope of the original meta-analysis, broad search terms were intentionally retained to capture all potentially relevant articles. Nevertheless, it likely that some relevant articles were missed; additionally, we excluded several relevant articles due to insufficient statistical information. Despite this, our final database included 251 articles drawn from 718 unique RCTs. We believe this is the largest effort to date synthesizing psychotropic medication effects on SITBs. Given that detected effects were largely consistent across potential moderators, we reason that the potential omission of a few studies would not meaningfully alter findings.

## Conclusions

To summarize, a small number of psychotropic medications were efficacious for a few specific SITB outcomes, while most medications did not significantly reduce SITBs. Our results are sobering, but they are consistent with prior reviews [28–31]. Due to the methodological constraints of the literature, it is possible that actual treatment effects are larger than those reported in RCTs. It is particularly notable that nearly half of included studies excluded participants on the basis of SITB risk, and less than 4% required SITBs for inclusion. Intentionally including individuals with SITBs in medication trials is critical for improving our understanding of psychotropic effects on SITBs. Future studies are also encouraged to advance the understanding of psychotropic medication mechanisms and the etiology of SITBs to improve pharmacological interventions for SITBs.

## Supplementary information

Supplemental Material
