# Supplementary material for: Efficacy of psychotropic medications on suicide and self-injury: a meta-analysis of randomized controlled trials
Source: Transl Psychiatry. 2022 Sep 21;12:400. doi: 10.1038/s41398-022-02173-9 (PMC9492722; doi:10.1038/s41398-022-02173-9)
Supplement: Supplementary file 1 — Supplemental Material [file 41398_2022_2173_MOESM1_ESM.docx]

**Supplemental Materials**

Supplementary Table S1. Additional Moderator Analyses for Specific Medication Classes, Stratified by Control Group Type

|  | **Binary Outcomes** | | | | |  |  |
| --- | --- | --- | --- | --- | --- | --- | --- |
| **Medication Class** | **Active Treatment** | |  | **Placebo** | |  |  |
|  | OR | 95% CI |  | OR | 95% CI |  |  |
| Antidepressants |  |  |  |  |  |  |  |
| SSRIs | 0.97 | [0.81, 1.17] |  | 0.96 | [0.84, 1.11] |  |  |
| SNRIs | 1.02 | [0.93, 1.12] |  | 0.93 | [0.78, 1.10] |  |  |
| Multimodal serotonin antagonists | 1.03 | [0.88, 1.19] |  | 0.88 | [0.78, 0.99] |  |  |
| NMDA receptor antagonists | 0.89 | [0.73, 1.10] |  | 0.69 | [0.46, 1.03] |  |  |
| Tricyclics and tetracyclics | - | - |  | 0.99 | [0.80, 1.22] |  |  |
| NaSSAs | - | - |  | 1.87 | [0.41, 8.50] |  |  |
| Antipsychotics |  |  |  |  |  |  |  |
| Atypical antipsychotics | 0.79 | [0.74, 0.85] |  | 0.94 | [0.81, 1.08] |  |  |
| Psychostimulants for ADHD and narcolepsy | - | - |  | 1.51 | [0.88, 2.60] |  |  |
| Agents for treatment of substance use disorders | 0.87 | [0.63, 1.21] |  | 0.76 | [0.44, 1.31] |  |  |
| Mood stabilizers | 0.89 | [0.65, 1.21] |  | 0.90 | [0.48, 1.70] |  |  |
| Agents for treatment of insomnia | 0.90 | [0.58, 1.38] |  | 1.05 | [0.46, 2.36] |  |  |
| Nonpsychostimulants for ADHD | - | - |  | 0.93 | [0.40, 2.18] |  |  |
| Anxiolytics | 0.81 | [0.20, 3.33] |  | - | - |  |  |

|  | **Continuous Outcomes** | | | | |  |  |
| --- | --- | --- | --- | --- | --- | --- | --- |
| **Medication Class** | **Active Treatment** | |  | **Placebo** | |  |  |
|  | *g* | 95% CI |  | *g* | 95% CI |  |  |
|  |  |  |  |  |  |  |  |
| Antidepressants |  |  |  |  |  |  |  |
| SSRIs | - | - |  | -0.17 | [-0.29, -0.05] |  |  |
| Antipsychotics |  |  |  |  |  |  |  |
| Atypical antipsychotics | -0.06 | [-0.19, 0.07] |  | -0.51 | [-1.07, 0.04] |  |  |
